# Lanthanide-doped bismuth-based nanophosphors for ratiometric upconversion optical thermometry†

**DOI:** 10.1039/d2ra01181f

**Published:** 2022-03-21

**Authors:** Jun Du, Jinliang Liu, Ying Chen, Yuefeng Zhao, Yuhao Li, Yuqing Miao

**Affiliations:** School of Materials and Chemistry, Institute of Bismuth and Rhenium Science, University of Shanghai for Science and Technology Shanghai 200093 China yhli@usst.edu.cn yqmiao@usst.edu.cn; Shanghai Collaborative Innovation Center of Energy Therapy for Tumors, University of Shanghai for Science and Technology Shanghai 200093 China

## Abstract

Nanothermometry could realize stable, efficient, and noninvasive temperature detection at the nanoscale. Unfortunately, most applications of nanothermometers are still limited due to their intricate synthetic process and low-temperature sensitivity. Herein, we reported a kind of novel bismuth-based upconversion nanomaterial with a fast and facile preparation strategy. The bismuth-based upconversion luminophore was synthesized by the co-precipitation method within 1 minute. By optimizing the doping ratio of the sensitizer Yb ion and the activator Er ion and adjusting the synthetic solvent strategy, the crystallinity of the nanomaterials was increased and the upconversion luminescence intensity was improved. Ratiometric upconversion optical measurements of temperature in the range of 278 K to 358 K can be achieved by ratiometric characteristic emission peaks of thermally sensitive Er ion. This method of rapidly constructing nanometer temperature probes provides a feasible strategy for the construction of novel fluorescent temperature probes.

## Abstract 2

Lanthanide-doped bismuth-based nanospheres can be rapidly synthesized within 1 minute for upconversion luminescence ratiometric temperature detection.

## Graphical abstract

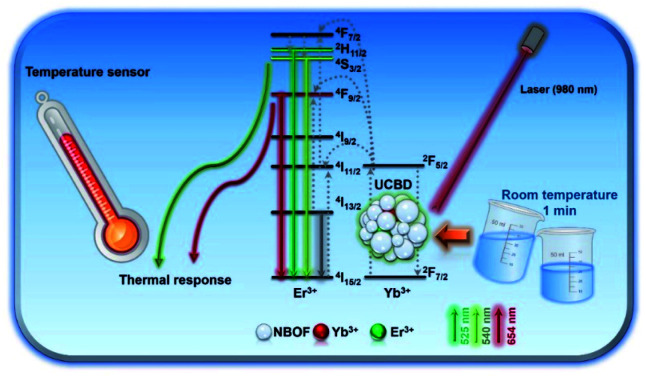

## 1 Introduction

Nanothermometry has been applied to a diversity of fields, including temperature detection within cells, diagnosis of microelectronics failures, application of nanorobots and so forth.^1–4^ Among a variety of nanothermometry techniques, fluorescent thermometers supply efficient and noninvasive approaches to measure *via* their nanoscale spatial resolution and marvelous optical properties.^5–8^ The ratio-type fluorescent probe for temperature detection has been widely reported because of its advantages of good temperature resistance, temperature resolution precision, response time, and anti-interference ability.^9^ Moreover, this method is more stable than other non-contact detection methods because lots of non-temperature factors are difficult to interfere with it during the temperature detection.^1,2,10^ In spite of the early achievement, it is significant to the field of nanothermometry to undergo evolution to provide methods with desirable sensitivity, dynamic range, and superior photostability.^1,2,11^

To date, a multitude of optical temperature sensing technologies has been explored on the basis of lanthanide-doped nanocrystals (Ln-NCs), the spectral diversity of which provides more options for the application of nanothermometry.^1,2,6,12–16^ It is likely to synthesize monodisperse nanoparticles with special morphology and optical uniformity *via* control of the growth of Ln-NCs.^17–24^ Generally, two fluorescence intensity ratio (FIR) from a single nanothermometer based on the coupled Ln-NCs energy states takes the unique advantage to accomplish the process of temperature sensing.^1,2,22^ With the use of Boltzmann thermal equilibrium, it is easy to calculate the temperature by FIR.^1,2,11,25–30^

The host materials of Ln-NCs are of a large variety, such as yttrium-based, gadolinium-based, lanthanum-based host materials and so on.^1,31,32^ For example, Mi *et al.* synthesized yttrium-based host materials by the hydrothermal method.^1^ Nevertheless, the synthetic process of the traditional host materials of Ln-NCs was too complicated to be beneficial to the preparation of Ln-NCs, most of which required high temperatures and took a long time to produce with low yield.^32,33^ Then Yi *et al.* reported the method of complex coprecipitation for the synthesis of yttrium-based hosts,^34^ but the process of after treatment remained to be simplified.^32^ Afterward, some research on bismuth-based hosts of Ln-NCs with a facile synthesis emerged.^32^ For instance, Trave *et al.* prepared NaBiF_4_ with a dopant of lanthanide ions at room temperature, and Back *et al.* reported rare-earth doped BiF_3_ likewise.^32^ There is no denying that the emergence of bismuth-based hosts could facilitate the large-scale production of Ln-NCs, however, the study on bismuth-based hosts of Ln-NCs with a simple method might be rare.

**Scheme 1:**
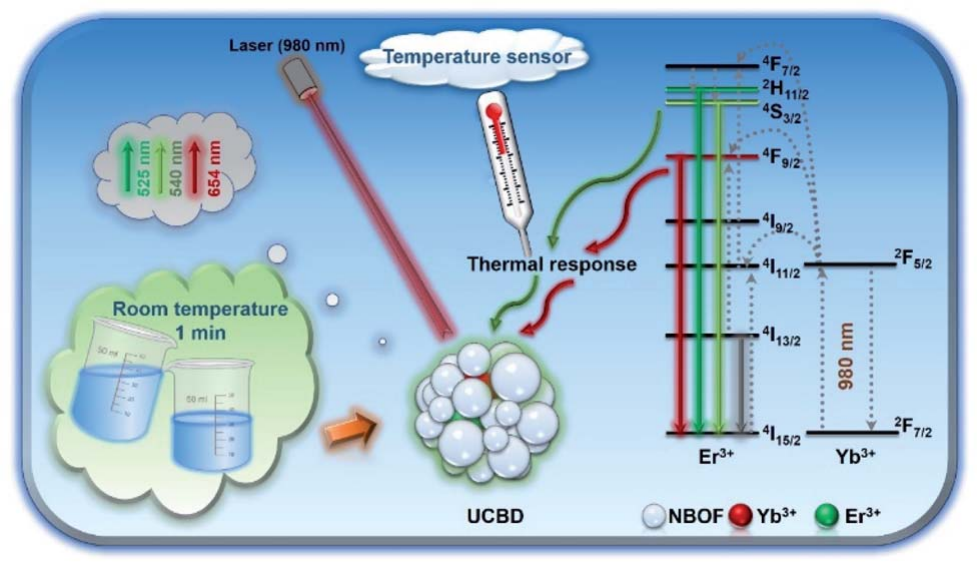
Schematic diagram of UCBD nanoparticles used in temperature-responsive ratiometric nanothermometers.

In our previous work, we also found a facile method for the rapid synthesis of bismuth-based rare-earth upconversion nanomaterial (Na_0.20_Bi_0.80_O_0.35_F_1.91_:Yb,Tm).^35,36^ This provides an effective and feasible way for the rapid fabrication of rare-earth upconversion luminescence nanothermometers. In the present work, we prepared bismuth-based upconversion luminescence nanoparticles doped with ytterbium and erbium ions (UCBE), which could be used in the area of nanothermometry (Scheme 1). By optimizing the preparation conditions, including adjusting the solvent, ion doping ratio, *etc.*, we found the upconversion luminescence property of the prepared bismuth-based upconversion nanomaterial (UCBD) was significantly improved, and it showed better sensitivity in ratiometric fluorescence thermometry. The exploration of this kind of material is beneficial to the development and application of new nanothermometers.

## 2 Results and discussion

### 2.1 Preparation and characterization of UCBD

Some bismuth-based compounds, including bismuth oxides and fluorides, can be rapidly synthesized by coprecipitation. Lanthanide ions with the same valence state can also be easily incorporated into bismuth-based nanomaterials.^35–37^ In our previous study, we rapidly synthesized a kind of mesoporous bismuth-based nanoparticles (Na_0.20_Bi_0.80_O_0.35_F_1.91_),^38–41^ however, its upconversion luminescence after doping with lanthanide elements is not ideal, and it cannot be used as a fluorescent temperature sensing probe. We speculated that the protic polar solvent ethylene glycol (EG) used in the synthesis may increase the surface defects of nanoparticles, resulting in unsatisfactory luminescence properties. Therefore, we adjusted the synthesis solvent and used dimethyl sulfoxide (DMSO, an aprotic polar solvent) as the solvent to synthesize bismuth-based nanoparticles to explore whether it could improve the luminescence ability. In addition, the characteristic emission peaks of erbium ion are sensitive to temperature, and it was doped to construct a fluorescence temperature nano-sensor.

**Fig. 1:**
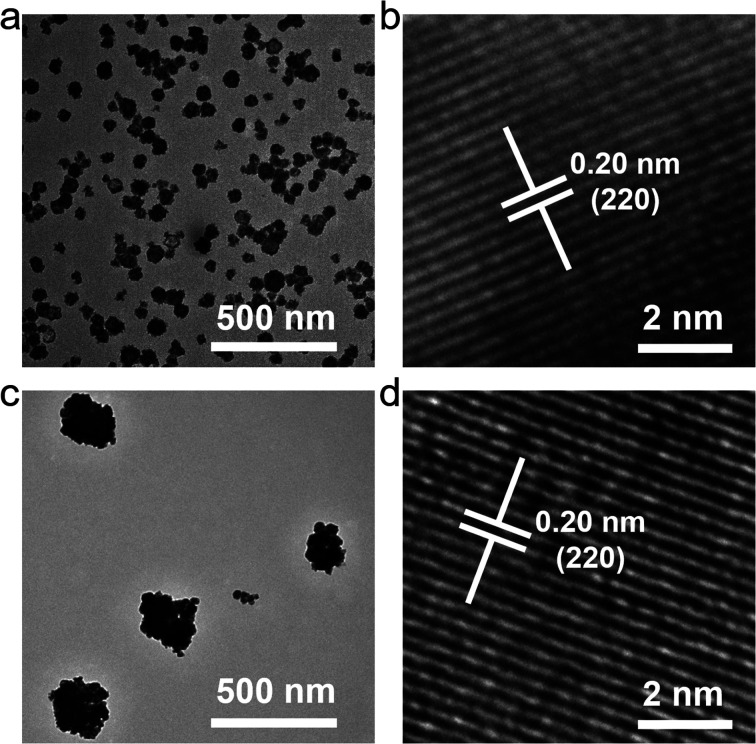
Morphology of UCBD. TEM images of the Na_0.20_Bi_0.80_O_0.35_F_1.91_:10%Yb,2%Er prepared in (a) ethylene glycol and in (c) dimethyl sulfoxide. High resolution TEM images of the Na_0.20_Bi_0.80_O_0.35_F_1.91_:10%Yb,2%Er prepared in (b) ethylene glycol and in (d) dimethyl sulfoxide.

As shown in Fig. 1, two nanoparticles synthesized by using EG or DMSO as the solvent have similar morphologies, all of which are spherical, indicating that the change of the solvent has little effect on the morphology. A single nanoparticle is composed of many smaller nanocrystals, which might be due to the addition of polyvinylpyrrolidone (PVP) to provide a template for the construction of nanocrystal. However, the particle size changed after the solvent was changed, and the nanoparticles synthesized in DMSO had a lager particle size with an average particle size of about 250–300 nm. In the high-resolution transmission electron microscope (TEM) results, the nanomaterials synthesized by the two solvents have the same lattice spacing of 0.20 nm, indicating that changing the solvent does not affect the lattice arrangement of the material.

**Fig. 2:**
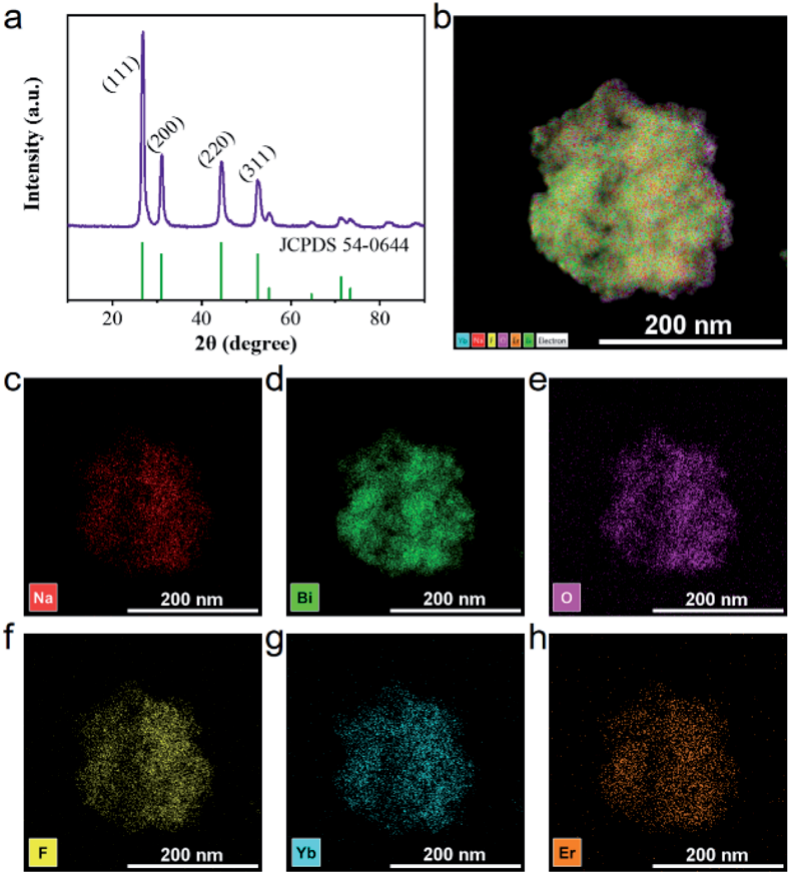
Structure of UCBD. (a) XRD. (b–h) EDS map scanning of UCBD and the corresponding element mappings of Na, Bi, O, F, Yb, and Er.

As shown in Fig. 2a, there are multiple characteristic peaks in the X-ray diffraction (XRD) pattern of UCBD such as 2*θ* = 26.86, 31.12, 44.46, and 52.86, which matched well with the peaks in the standard diffraction card (JCPDS card no. 54-0644), indicating that the material synthesized in DMSO was still Na_0.20_Bi_0.80_O_0.35_F_1.91_. However, due to the doping of Yb and Er elements, the experimental peak 2*θ* value is slightly shifted to a higher direction compared with the peak in the standard card. In addition to these main peaks, no other peaks were found in the XRD pattern, indicating that the synthesized material was of good purity. The 2*θ* (2.04 Å) corresponding to the crystal planes (220) observed in XRD match well with the observed lattice spacing (0.20 nm), which also presented that the synthesized material is Na_0.20_Bi_0.80_O_0.35_F_1.91_:Yb,Er. The element distribution of the UCBD was then analyzed (Fig. 2b–h and S1†), and six elements (Na, Bi, O, F, Yb, and Er) were observed from energy disperse spectroscopy (EDS) mapping and uniformly dispersed in the nanomaterial, indicating that it is feasible to replace bismuth with lanthanide ions of the same valence.

**Fig. 3:**
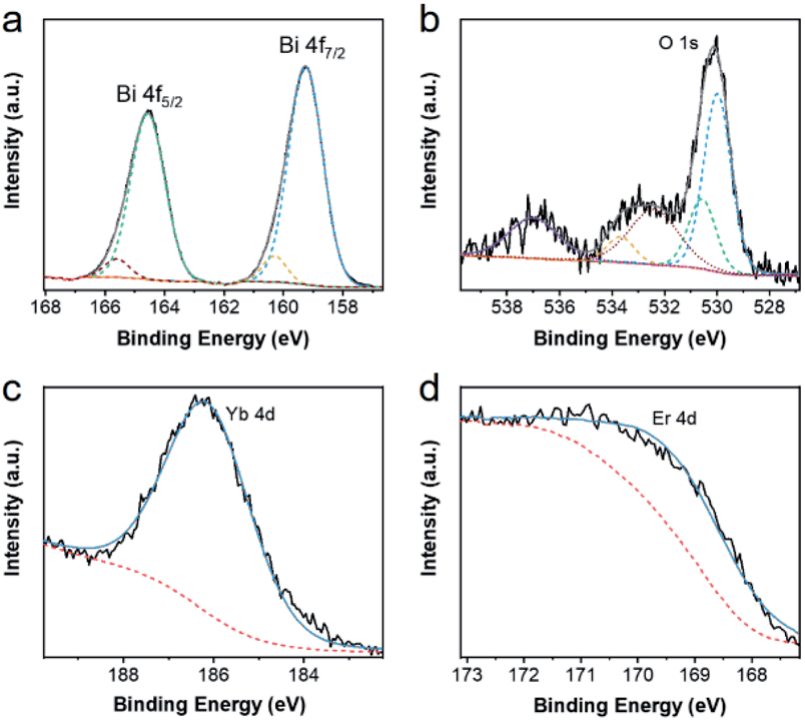
High-resolution XPS spectra of (a) Bi 4f, (b) O 1s, (c) Yb 4d, and (d) Er 4d in UCBD.

Then, X-ray photoelectron spectroscopy (XPS) was performed to confirm the elemental chemical states of the as-synthesized products,^42^ which were demonstrated in Fig. 3 and S2.† After using the Gauss–Lorentz curve fitting, several peaks could be separated. In Fig. 3a, two strong peaks centered at 159.26 eV and 164.57 eV, corresponding to the Bi 4f_7/2_, and Bi 4f_5/2_ respectively, the difference between which was 5.31 eV, were ascribed to the Bi–O bond. Meanwhile, two other peaks located at 160.31 eV and 165.62 eV, corresponding to the Bi 4f_7/2_ and Bi 4f_5/2_, could result from the Bi–F bond, and the difference between them was also 5.31 eV. The high-resolution spectra of O 1s in Fig. 3b could be decomposed into five peaks. The band of 530.01 eV was assigned to the Bi–O bond, the band of 530.56 eV was attributed to the Yb–O bond, and the band of 533.71 eV might stem from humid atmospheres. Moreover, the band of 532.41 and that of 537.01 could be ascribed to Na KLL peaks. In Fig. 3c and d, the peak position centered at 186.26 eV was Yb 4d peak. Similarly, the peak of Er 4d was located at 169.41 eV. The high-resolution spectra of F 1s could be divided into two peaks and the peak position centered at 684.51 eV could be attributed to F 1s, while that of 680.96 eV could be ascribed to Bi 4p_3/2_. Furthermore, the high-resolution spectra of Na 1s and Er 4p are located at 1071.66 eV and 320.31 eV respectively. All of these characterizations demonstrated the elemental composition and valence state of UCBD.

### 2.2 Photoluminescence properties of UCBD

**Fig. 4:**
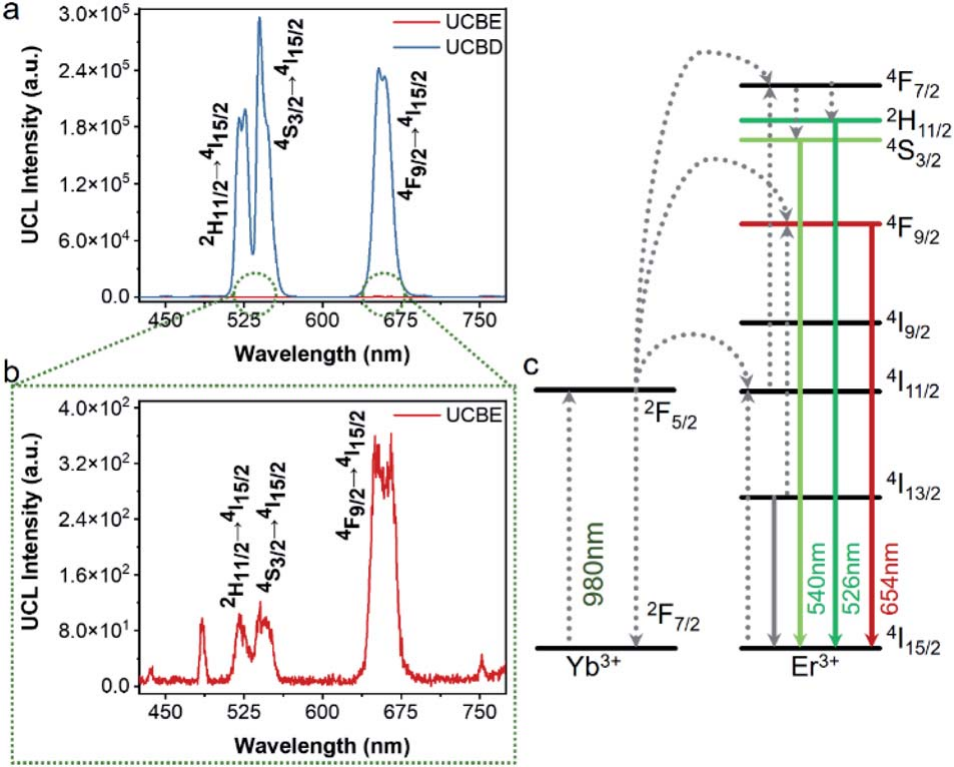
Photoluminescence properties of UCBD and UCBE doped with 10% Yb and 2% Er. (a) Upconversion emission spectra of UCBE and UCBD. (b) The enlarged emission spectrum of UCBE. (c) Schematic illustration of the energy transfer pathways of Yb^3+^ and Er^3+^ ions under 980 nm excitation.

Next, the luminescence properties of UCBD and UCBE were investigated. As shown in Fig. 4a and b, under the excitation of 980 nm light, they both exhibited three emission peaks in the range of 500–680 nm, and the main peaks were located at 526 nm, 542 nm, and 654 nm, which could be attributed to the characteristic emission peaks of Er ion. This indicates that Er ion can also achieve upconversion luminescence in the bismuth-based nanomatrix.^10,43^ According to the previous research mechanism (Fig. 4c), Yb^3+^ acts as a sensitizer to absorb the energy of 980 nm light in the upconversion luminescent material and then transfers it to the activator Er^3+^ ion.^43,44^ Due to the single-photon, two-photon, or multi-photon energy transfer process, Er^3+^ ion with abundant outer electrons return to the ground state at different electronic excited state energy levels and emit characteristic emission peaks. The three characteristic emission peaks we observed can be assigned to ^2^H_11/2_ → ^4^I_15/2_ transitions, ^4^S_3/2_ → ^4^I_15/2_ transitions, and the ^4^F_9/2_ → ^4^I_15/2_ transitions,^1,32,35^ respectively. However, what excited us most was that the fluorescence intensity from UCBD was more than a hundred times higher than that in UCBE, which might be caused by higher crystallinity.

**Fig. 5:**
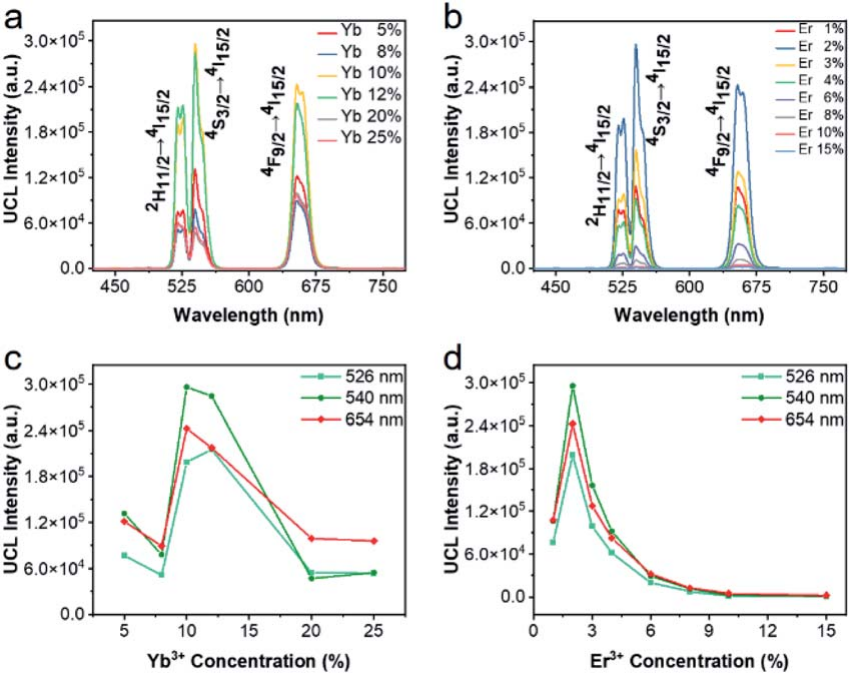
Photoluminescence properties of UCBD. (a and c) Emission spectra of UCBD with 2% Er^3+^ dopant and different Yb^3+^ dopant from 5% to 25%. (b and d) Emission spectra of UCBD with 10% Yb^3+^ dopant and different Er^3+^ dopant from 1% to 15%.

To obtain the brightest upconversion luminescence in UCBD, we adjusted the doping content of ytterbium and erbium ions, as exhibited in Fig. 5a and b. It was obvious that the luminescence intensity of UCBD firstly increased and then decreased with the increase of ytterbium and erbium ions doping concentration. When the concentration of ytterbium ion was lower than 6%, the number of sensitizers in UCBD was not enough, which could not effectively excite the luminescent center leading to the decrease of luminescent efficiency (Fig. 5c). When the doping concentration of ytterbium ion was higher than 20%, the distance between ytterbium and erbium ions was shortened, so that the energy of erbium ion was transferred back to ytterbium ion in the form of cross-relaxation resulting in concentration quenching, which induced a significant decrease in the luminescent intensity of UCBD. Therefore, the best doping content of ytterbium ion was selected at 10%. Likewise, Fig. 5d indicated when the doping concentration of erbium ion was less than 1%, the number of luminescence centers in UCBD was not abundant leading to the decline of the luminescence efficiency. When the doping concentration of erbium ion was higher than 2%, the distance between erbium ion was shortened, so that there was cross-relaxation between neighboring erbium ions, and the energy was consumed in the mutual transfer between erbium ions resulting in concentration quenching, which caused an obvious decrease in the luminescent intensity. So, the optimal doping content of erbium ion was selected at 2%.

### 2.3 Optical thermometry behavior of UCBD

**Fig. 6:**
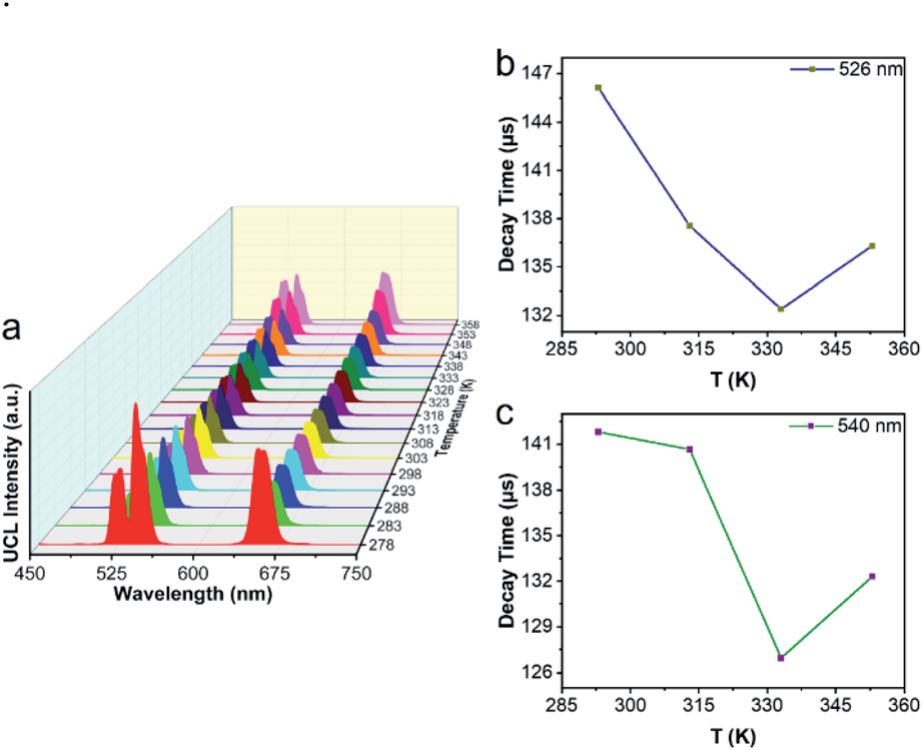
Thermal response of UCBD doped with 10% Yb and 2% Er. (a) Emission spectra of UCBD excited by 980 nm light at a different temperature from 278 K to 358 K with a 5 K temperature interval. The average luminescence lifetime is at (b) 526 nm and (c) 540 nm.

Next, we used UCBD, doped with 10% Yb^3+^ and 2% Er^3+^ synthesized under optimized conditions to explore its performance as a temperature fluorescence nano-sensor. The emission spectra of UCBD at temperature were shown in Fig. 6a. When the temperature gradually rose from 278 K to 318 K, the intensity of emission peak centered at 526 nm decreased conspicuously, which was induced by the thermal quenching from ^2^H_11/2_ → ^4^I_15/2_ transition.^1,10^ However, with the increase of temperature from 318 K to 358 K, its intensity presented an obvious trend of increase, which was due to the thermal enhancement of ^2^H_11/2_ → ^4^I_15/2_ transition.^1,10^ Similarly, while the temperature increasingly rose from 278 K to 333 K, the emission peak strength centered at 540 nm declined due to the thermal quenching from ^4^S_3/2_ → ^4^I_15/2_ transition.^1,10^ By contrast, with the rise of temperature from 333 K to 358 K, its strength increased because of the thermal enhancement of ^4^S_3/2_ → ^4^I_15/2_ transition.^1,10^

To make a further study on thermal-responsive behavior of UCBD, the decay time of 526 nm, 540 nm, and 654 nm emission peaks at different temperatures was shown in Fig. S3.† While the average lifetime was calculated by the formula (1), in which *t* was time and *I*(*t*) referred to the fluorescence intensity at *t*.^2,11^1
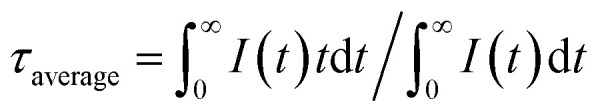


As shown in Fig. 6b, the average lifetime of 526 nm emission at the temperature of 293 K, 313 K, 333 K, and 353 K was 146.12 μs, 137.55 μs, 132.39 μs, and 136.31 μs respectively. While Fig. 6c showed the average lifetime of 540 nm emission was 141.81 μs, 140.66 μs, 126.94 μs, and 132.31 μs correspondingly. In addition, as demonstrated in Fig. S3,† the average lifetime of 654 nm emission was 415.27 μs, 397.05 μs, 384.63 μs, and 379.16 μs at the same temperature. As a result, the emission lifetime also changed with temperature, which suggested that the UCBD was of great potential in nanothermometry due to its thermal response.

**Fig. 7:**
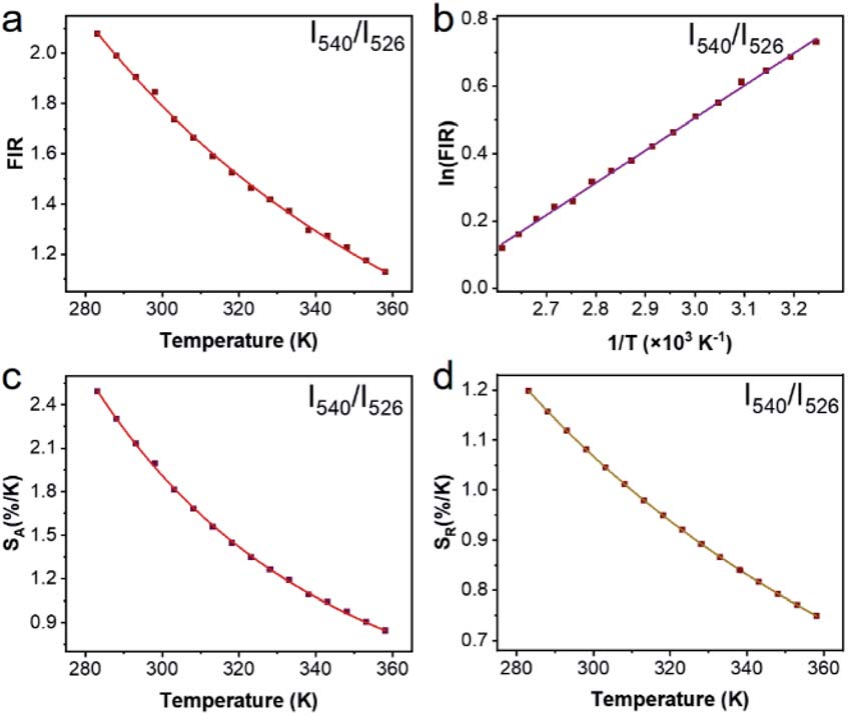
Properties of the ratiometric nanothermometers. (a) Fluorescence intensity ratios (FIR) of UCBD were calculated by emission at 540 nm and 526 nm. (b) ln(FIR) of UCBD calculated by (a). (c) Absolute sensitivities of the nanothermometers based on the FIR in (b). (d) Relative sensitivities of the nanothermometers on basis of the FIR in (b).

To justify whether the UCBD could apply to actual temperature measurement, it was essential to use the technology of FIR. A simplified model of Boltzmann thermal equilibrium, as shown in formula (2), was used to describe the FIR technology^1,2,25,45,46^2
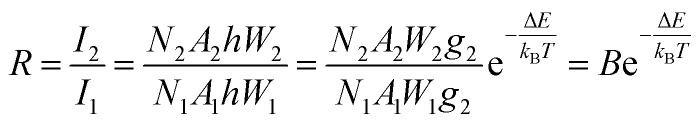
in which *I*_2_ and *I*_1_ were the intensities of 526 and 540 nm emission from the transitions of ^2^H_11/2_ → ^4^I_15/2_ and ^4^S_3/2_ → ^4^I_15/2_ respectively. *N*_2_ and *N*_1_ were the populations at the ^2^H_11/2_ and ^4^S_3/2_ levels, and *A*_2_ and *A*_1_ were the total spontaneous-emission rates of the ^2^H_11/2_ and ^4^S_3/2_ levels transfer to ground state, while *g*_2_, *g*_1_, and *ω*_2_, *ω*_1_ were the degeneracy and the angular frequency of the ^2^H_11/2_ and ^4^S_3/2_ levels, respectively. Δ*E* and *T* represented the energy gap between the ^2^H_11/2_ and ^4^S_3/2_ levels and the absolute temperature, correspondingly. As illustrated in Fig. 7a, the emission intensity of 526 nm and 540 nm was selected to calculate FIR values by formula (2). Fig. 7b showed the values of ln(FIR) calculated from Fig. 7a. And the FIR and ln(FIR) values calculated from the 540 nm and 654 nm emission intensity were also shown in Fig. S4a and b.† In addition, thermal sensitivity referred to the rate of change of temperature measurement parameters with temperature changes. In practical application, the absolute thermal sensitivity (*S*_a_) and the relative temperature sensitivity (*S*_r_), significant parameters for temperature detection, were expressed as3
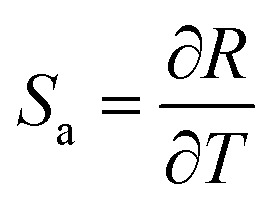
4
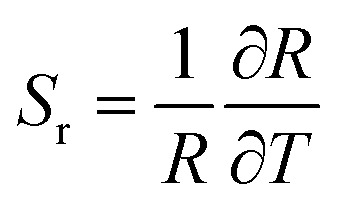
depending only on the degree of the thermally induced variations in *R*.^1,2,25,45–47^ The curve of *S*_a_ and *S*_r_ of *I*_540_/*I*_526_ with the change of temperature demonstrated in Fig. 7c and d suggested that *S*_a_ and *S*_r_ decreased monotonously, reaching the maximum value of 2.49 K^−1^ and 1.24 K^−1^ respectively at 283 K. However, the calculated *S*_a_ and *S*_r_ of *I*_540_/*I*_526_ could reach the maximum value of 0.25 K^−1^ and 0.30 K^−1^ respectively at 283 K (Fig. S4c and d†). For the *S*_r_ value of *I*_540_/*I*_526_, it was conspicuous that the thermal sensitivity of UCBD seemed higher than those previously reported in Table S1† based on FIR technology. Moreover, temperature resolution (δ*T*), another crucial parameter for temperature measurement, was a symbol of the minimum temperature resolvable by the thermometer, and it only depended on the material and the experimental setup used, described by the following equation.5
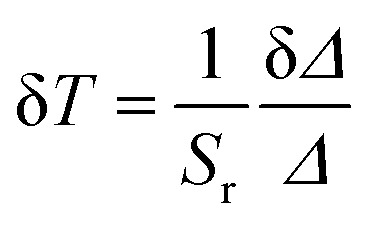
in which δ*Δ*/*Δ* refers to the relative uncertainty in the measurement of temperature measurement parameters.^1,2,48^ This depends on the acquisition settings and is estimated from the error in *Δ*. In this case, the average value of δ*Δ*/*Δ* is about 0.5%. Hence, as described in Fig. 7d, the minimum value of temperature resolution at 283 K was 0.40 K. At last, to detect the temperature sustainably, thermal repeatability was necessary. According to the equation of the multiple-cycle test, the temperature measurement repeatability could be quantified.6
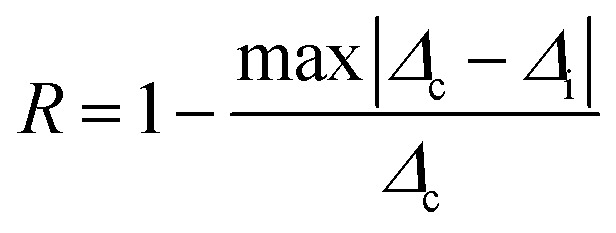
where *Δ*_c_ was the mean thermometric signal from different cycles and *Δ*_i_ was the thermometric signal in each cycle.^1,2,45^

**Fig. 8:**
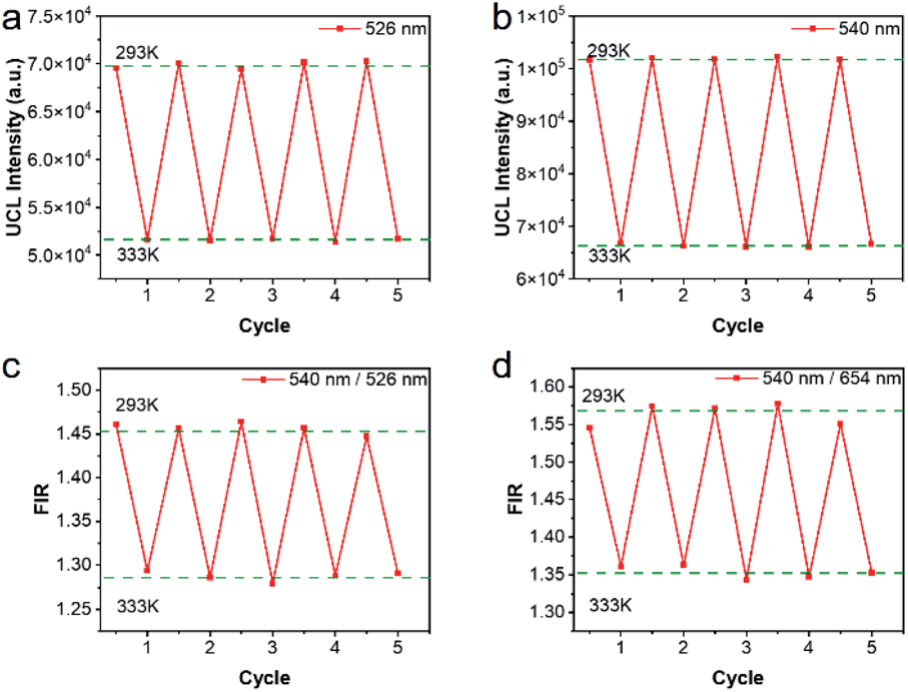
Repeatability of the nanothermometer in 40 K heating–cooling cycles. The emission intensities of 526 nm (a) and 540 nm (b) including the corresponding FIRs (c) and (d) were recorded from 293 to 333 K at each cycle.

Repeatability is also an important indicator for evaluating temperature probes. The curves of 5 heating–cooling cycle tests were described in Fig. 8 and S5.† After multiple thermal cycles, the emission intensity at a single wavelength such as 526 nm, 540 nm, and 654 nm, and the ratio of the two emission intensities at 540 nm/526 nm and 540 nm/654 nm remained basically unchanged at 293 K and 333 K, showing high reproducibility. By calculation, as illustrated in Fig. 8c and d, the repeatability ranged from 99.4% to 99.9%, indicating the reliability of the ratiometric thermometers.

## 3 Experimental section

### 3.1 Materials

All chemicals were purchased from commercial sources without any further purification. Bismuth nitrate pentahydrate (Bi(NO_3_)_3_·5H_2_O, 99.9%, Shanghai Chemical Reagent, China), ytterbium nitrate pentahydrate (Yb(NO_3_)_3_·5H_2_O, 99.99%, Sigma, USA), erbium nitrate hexahydrate (Er(NO_3_)_3_·6H_2_O, 99.99%, Sigma, USA), sodium nitrate (NaNO_3_, 99.9%, Adamas, China), PVP (K30, Adamas, China), ammonium fluoride (NH_4_F, 98%, Aladdin, China), dimethyl sulfoxide (99.0%, General-reagent, China), ethylene glycol (99%, General-reagent, China).

### 3.2 Synthesis of UCBD or UCBE

Yb(NO_3_)_3_·5H_2_O (*x* mmol, *x* = 0.05, 0.08, 0.10, 0.12, 0.15, 0.20, and 0.25), Er(NO_3_)_3_·6H_2_O (*y* mmol, *y* = 0.01, 0.02, 0.03, 0.04, 0.06, 0.08, 0.10, and 0.15), Bi(NO_3_)_3_·5H_2_O (1 − *x* − *y* mmol), NaNO_3_ (2 mmol), and PVP (0.4 g) were dissolved in 20 mL of solvent (EG for UCBE or DMSO for UCBD) to form a kind of transparent solution A. And NH_4_F (4 mmol) was dissolved in 15 mL of solvent (EG or DMSO) to form another kind of solution B. Subsequently, solution B was poured into solution A under vigorous stirring for 60 s at room temperature. The reaction was quenched with an equal volume of H_2_O (35 mL). Next, the precipitation was collected through centrifugation at 15 000 rpm for 10 min and washed three times with ethanol. At last, some final products were dispersed and kept in ethanol, and the others were dried into powder for further use.

### 3.3 Characterization techniques

TEM images and EDS mappings and spectrum were captured by FEI Tecnai F20. XRD was measured on the Bruker D8 instrument. XPS spectra were acquired from Thermo Scientific K-Alpha.

### 3.4 Measurement of optical property

Upconversion emission spectra and lifetime were obtained from a fluorescence spectrometer (Edinburgh FS5, UK), equipped with an adjustable 980 nm laser (MDL-III-980-2W, CNI, China). 0.75 W of power was used to excited the UCBD to emit upconversion emission. The temperature-dependent measurements in the air were performed by using a temperature control device (TC1 temperature controller, Quantum Northwest, USA). The temperature could be controlled from 278 K to 358 K with increments down to 0.1 K. All of the temperature-dependent spectra detection of UCBD was performed in a powder state.

## 4 Conclusions

In summary, we synthesized a kind of novel bismuth-based upconversion nanoparticles UCBD with a facile strategy. The reaction was completed very fast and convenient within only 1 min at room temperature. After changing the solvent from EG to DMSO, the fluorescence intensity was enhanced more than a hundred times. Then the optical property was further optimized by adjusting the doping content of ytterbium and erbium ions. The upconversion luminescence in UCBD was brightest when the doping content of ytterbium ions and erbium ions were selected at 10% and 2% respectively. Furthermore, we made a study on the optical thermometry behavior of UCBD, confirming the thermal-responsive performance. Finally, a kind of ratiometric thermometer was constructed by using FIR technology. The maximum value of *S*_a_ and *S*_r_, calculated from FIR at the temperature of 283 K, was 2.49 K^−1^ and 1.24 K^−1^ respectively. The temperature resolution calculated was 0.40 K, and the repeatability ranged from 99.4% to 99.9%, confirming the reliability of the ratiometric thermometers. On balance, UCBD is of great potential to be applied in temperature detection *via* its unique advantages above, mainly including the fast and facile strategy for synthesis, the enhancement of fluorescent, and the temperature-responsive property.

## Author contributions

This manuscript was written and approved by all authors.

## Conflicts of interest

There are no conflicts to declare.

## Supplementary Material

RA-012-D2RA01181F-s001
