# Supplementary material for: Lanthanide-doped bismuth-based nanophosphors for ratiometric upconversion optical thermometry
Source: RSC Adv. 2022 Mar 21;12(14):8743–9. doi: 10.1039/d2ra01181f (PMC8985227; doi:10.1039/d2ra01181f)
Supplement: RA-012-D2RA01181F-s001 [file RA-012-D2RA01181F-s001.pdf]

Electronic Supplementary Material (ESI) for RSC Advances.
This journal is © The Royal Society of Chemistry 2022

Supplementary data

Lanthanide-Doped Bismuth-Based Nanophosphors for Ratiometric Upconversion Optical Thermometry

Jun Du[a], Jinliang Liu[a], Ying Chen[a], Yuefeng Zhao[a], Yuhao Li[a,b,*], Yuqing Miao[a,b,*]

a. School of Materials and Chemistry & Institute of Bismuth and Rhenium Science, University of Shanghai for Science and Technology, Shanghai 200093, China. E-mail: yhli@usst.edu.cn

b. Shanghai Collaborative Innovation Center of Energy Therapy for Tumors, University of Shanghai for Science and Technology, Shanghai 200093, China

* Corresponding author

E-mail: yhli@usst.edu.cn (Y. Li), yqmiao@usst.edu.cn (Y. Miao)

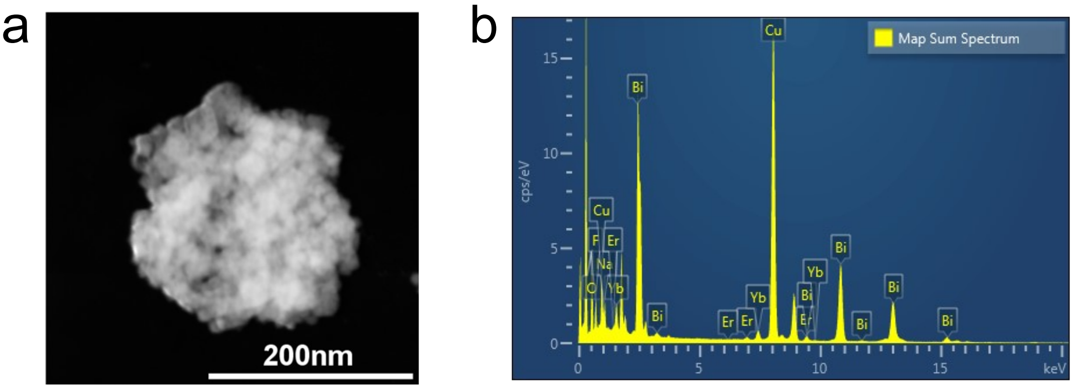

Fig. S1 HAADF-STEM image (a) and EDS spectrum (b) of UCBD.

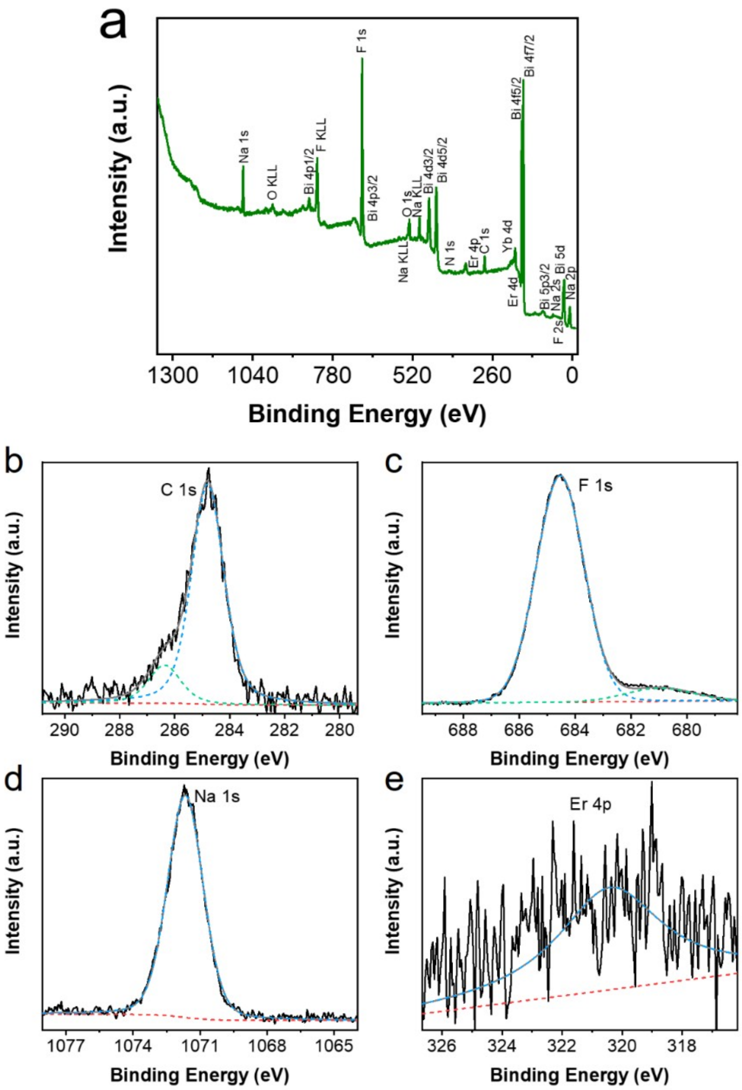

Fig. S2 XPS of UCBD doped with 10% Yb and 2% Er (a) and High-resolution XPS of C 1s (b), F 1s (c), Na 1s (d), and Er 4p (d) in UCBD.

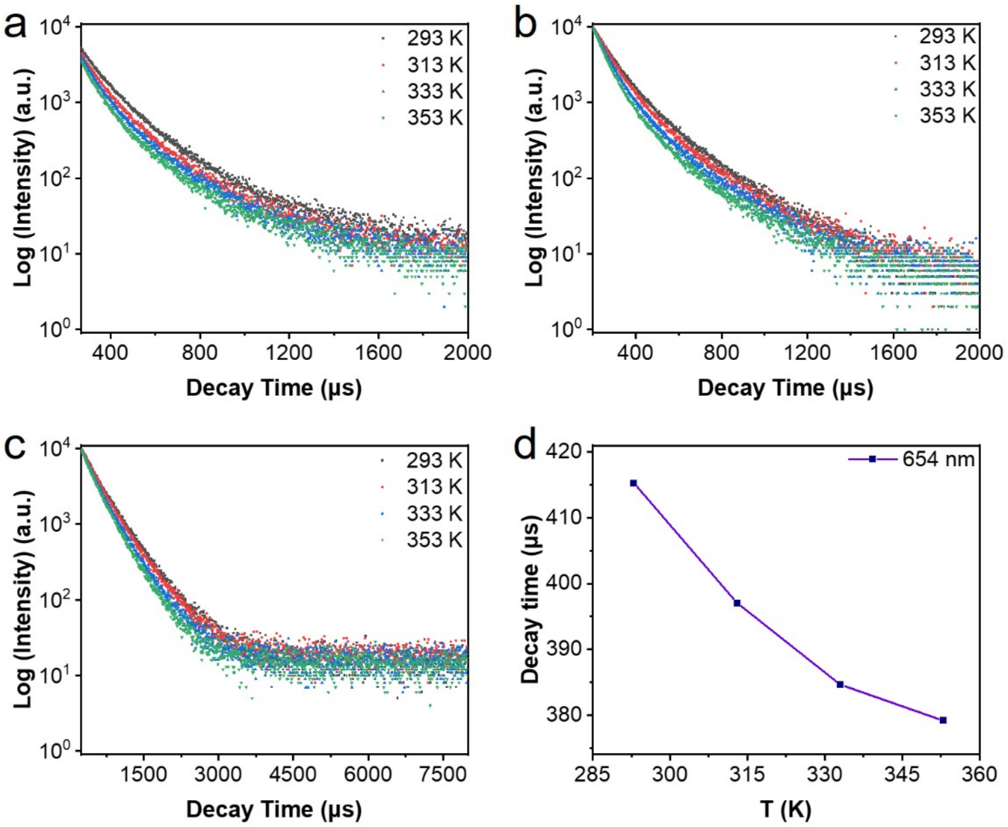

Fig. S3 Emission lifetime at 526 nm (a), 540 nm (b), and 654 nm (c) at different temperatures. (d) A plot of calculated average emission lifetime at 654 nm at different temperatures.

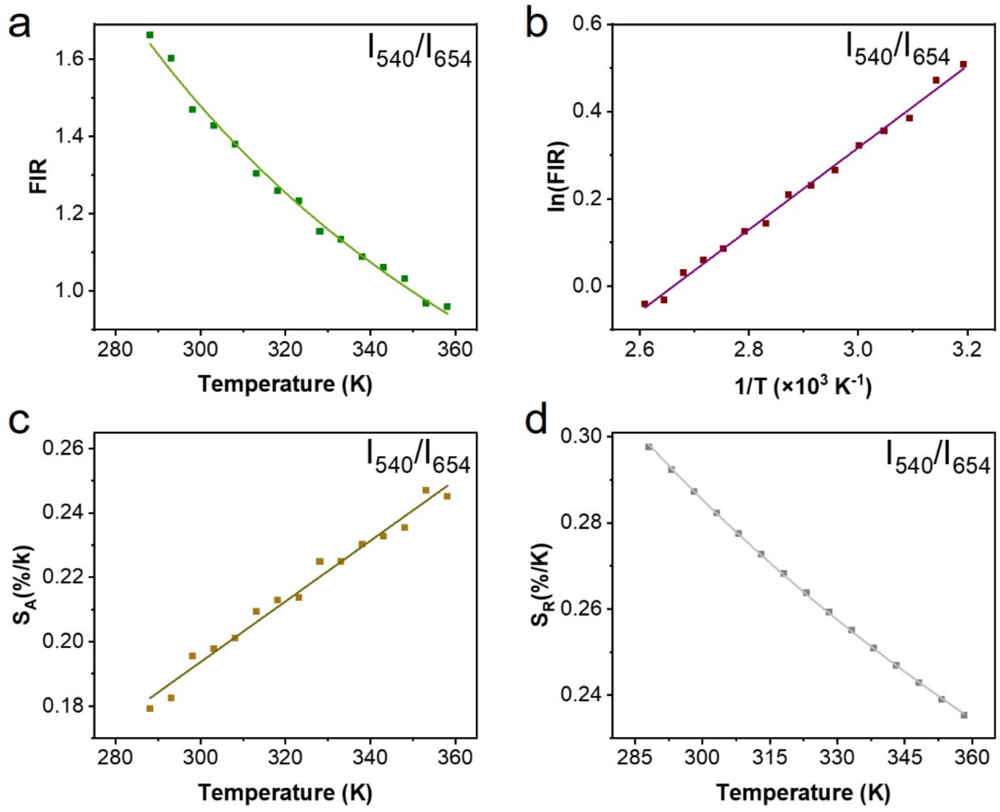

Fig. S4 Properties of the UCBD nanothermometers. (a) Fluorescence intensity ratios (FIRs) were calculated by 540 nm emission and 654 nm emission. (b) ln (FIR) calculated by (a). (c) Absolute sensitivities based on the FIRs in (b). (d) Relative sensitivities on basis of the FIRs in (b).

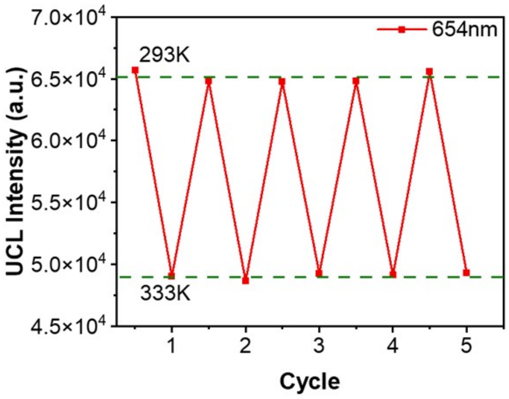

Fig. S5 Repeatability of the UCBD nanothermometer in 40 K heating−cooling cycles. The emission intensity of 654 nm was recorded from 293 to 333 K at each cycle.

Table S1 The comparison of Sr value of different nanomaterials.

Materials	Sr [% K^{-1}]	Reference
$Y_2O_3:Eu^{3+}$	7.8×10^{-2}	1
$Y_2O_3:Yb^{3+}/Ho^{3+}$	1.6	2
TiO2:Tm^{3+}	3.0×10^{-4}	3
$NaYbF_4:Tm^{3+}@SiO_2$	5.6×10^{-2}	4
$NaYF_4$: $Yb^{3+}/Er^{3+}@SiO_2$	1.0	5
$Y_2O_3:Tb^{3+}/Tm^{3+}$	0.33	6
$NaGdF_4:Er^{3+}/Ho^{3+}/Yb^{3+}@SiO_2$	1.1	7
$YVO_4:Nd^{3+}$	0.54	8
$Na_{0.20}Bi_{0.80}O_{0.35}F_{1.91}:Yb^{3+}/Er^{3+}$ (UCBD)	1.24	This work

Reference:

1. H. S. Peng, H. W. Song, B. J. Chen, J. W. Wang, S. Z. Lu, X. G. Kong and J. H. Zhang, *J. Chem. Phys.*, 2003, **118**, 3277–3282.
2. N. N. Dong, M. Pedroni, F. Piccinelli, G. Conti, A. Sbarbati, J. E. Ramirez-Hernandez, L. M. Maestro, M. C. Iglesias-de la Cruz, F. Sanz-Rodriguez, A. Juarranz, F. Chen, F. Vetrone, J. A. Capobianco, J. G. Sole, M. Bettinelli, D. Jaque and A. Speghini, *ACS Nano*, 2011, **5**, 8665–8671.
3. A. R. Zanatta, D. Scoca and F. Alvarez, *Sci. Rep.*, 2017, **7**, 14113.
4. X. F. Wang, J. Zheng, Y. Xuan and X. H. Yan, *Opt. Express*, 2013, **21**, 21596–21606.
5. R. G. Geitenbeek, P. T. Prins, W. Albrecht, A. van Blaaderen, B. M. Weckhuysen and A. Meijerink, *J. Phys. Chem. C*, 2017, **121**, 3503–3510.
6. N. Ishiwada, S. Fujioka, T. Ueda and T. Yokomori, *Opt. Lett.*, 2011, **36**, 760–762.
7. A. Skripka, A. Benayas, R. Marin, P. Canton, E. Hemmer and F. Vetrone, *Nanoscale*, 2017, **9**, 3079–3085.
8. I. E. Kolesnikov, A. A. Kalinichev, M. A. Kurochkin, E. V. Golyeva, E. Y. Kolesnikov, A. V. Kurochkin, E. Lahderanta and M. D. Mikhailov, *Sci. Rep.*, 2017, **7**, 18002.
